# A Link Between Attentional Function, Effective Eye Movements, and Driving Ability

**DOI:** 10.1037/xhp0000297

**Published:** 2016-11-28

**Authors:** Andrew K. Mackenzie, Julie M. Harris

**Affiliations:** 1 Division of Psychology, Nottingham Trent University, and School of Psychology & Neuroscience, University of St. Andrews; 2 School of Psychology & Neuroscience, University of St. Andrews

**Keywords:** eye movements, driving, visual attention, visual cognition

## Abstract

The misallocation of driver visual attention has been suggested as a major contributing factor to vehicle accidents. One possible reason is that the relatively high cognitive demands of driving limit the ability to efficiently allocate gaze. We present an experiment that explores the relationship between attentional function and visual performance when driving. Drivers performed 2 variations of a multiple-object tracking task targeting aspects of cognition including sustained attention, dual-tasking, covert attention, and visuomotor skill. They also drove a number of courses in a driving simulator. Eye movements were recorded throughout. We found that individuals who performed better in the cognitive tasks exhibited more effective eye movement strategies when driving, such as scanning more of the road, and they also exhibited better driving performance. We discuss the potential link between an individual’s attentional function, effective eye movements, and driving ability. We also discuss the use of a visuomotor task in assessing driving behavior.

Driving is a common everyday, yet complex, visuomotor task. It requires attention to the ever changing environment, to hazards that may appear, and to the control of the vehicle itself. Inattention and subsequent failures to scan the roadway are often reported as being contributing factors to vehicle accidents (Dingus et al., 2006; Klauer, Dingus, Neale, Sudweeks, & Ramsey, 2006; Lee, 2008). In this study, we explore how individual differences in visual cognition may correlate with effective visual behavior; eye movements that are typically associated with safer drivers. We hypothesize that competition for processing resources could limit efficient driving behavior and eye movement behavior. Therefore, we expect those who are better able to deploy attention will show more appropriate eye movement behavior. We also explore an interactive visual attention task to test the hypothesis that active visual attention tasks, requiring sustained attention, may be useful predictors of visual scanning behavior and driving performance. Before we describe our study, we outline some of the literature regarding eye movements and driving and discuss how an individual’s visual attention might relate to eye movement behavior and driving performance.

## Eye Movement Behavior: From Novice to Experienced

Many studies have measured eye movements during driving. Typically, drivers tend to fixate straight ahead when driving, usually to the location where the vehicle will be in the next few seconds, at least on straight and undemanding roads (Mourant & Rockwell, 1972; Underwood, Chapman, Brocklehurst, Underwood, & Crundall, 2003). Individuals will scan left and right of this point in space while driving, but the extent of this horizontal scanning can be different between novice and experienced drivers. Experienced drivers tend to exhibit a wider horizontal search strategy compared to novice drivers (Alberti, Shahar, & Crundall, 2014; Crundall & Underwood, 1998; Falkmer & Gregersen, 2001; Konstantopoulos, Chapman, & Crundall, 2010; Underwood, Chapman, Bowden, & Crundall, 2002). Crundall, Chapman, Phelps, and Underwood (2003) demonstrated this effect to be linked not only to experience (i.e., how long someone has driven) but also to expertise. They showed that police drivers, who are trained to be “expert” drivers, exhibit a wider search strategy relative to age matched and experience matched control drivers. This type of visual behavior is important because wider scanning may result in more peripheral hazards being detected for example, looking to the side pavements for possible pedestrians stepping out, inspecting slip roads more often for joining traffic or looking around for possible undertaking or overtaking vehicles in more demanding situations.

Two main suggestions have been proposed for why there are visual behavior differences between experienced and novice drivers. The first is the notion that novice drivers do not know where to look during driving, in that they are not aware of the potentially hazardous areas of the road. The second is that because the driving task is less automated for novice drivers, the majority of their attentional resources are given to vehicle control. They therefore lack the attentional resources to allocate visual attention to areas of the driving scene that are typically associated with safe driving. Note that these are not competing hypotheses as both likely contribute to differences in visual behavior.

The first hypothesis has been tackled by others, suggesting that some individuals lack a developed mental model of the situations that may be encountered on the road (Underwood, 2007; Underwood, Chapman, et al., 2002). In other words, they lack situation awareness (e.g. Endsley & Garland, 2000). Via experience of driving, with exposure to different situations and road users, drivers are thought to build up a mental model of the potential hazards that may arise on a given road type, and are able to allocate visual attention accordingly (see Endsley, 1995a, 1995b, 2004; Underwood, 2007; Underwood, Crundall, & Chapman, 2011; Wickens, 2008b).

Our study is focused on the alternative hypothesis. *Cognitive load* is a term used to infer the attentional demands of a task (Wickens, 2002, 2008a). The allocation of attentional resources during a task is largely affected by the level of cognitive load (Tomasi, Chang, Caparelli, & Ernst, 2007; Wickens & Hollands, 2000). Eye movements and attention are intrinsically linked (Corbetta et al., 1998; Klein, Kingstone, & Pontefract, 1992); thus, if there is interference with attentional deployment, eye movement behavior could change as a result. In driving, we know that increasing cognitive load during driving tasks (usually by introducing a secondary cognitive task) reduces horizontal scanning behavior and use of the mirrors (Engström, Johansson, & Östlund, 2005; Recarte & Nunes, 2003; Savage, Potter, & Tatler, 2013). In our previous work (Mackenzie & Harris, 2015), we directly compared individuals’ eye movement behavior during a passive video-based hazard perception task and an analogous, but more cognitively demanding, active driving task. We found that those who performed the active driving task scanned the roadway less than those who performed the passive task. Together, these studies suggest that increasing the cognitive demand during driving reduces the amount of attentional resources that can be allocated to visually scanning the road. And thus, these studies may provide indirect evidence for the idea that improvements in visual behavior with experience occur because the process of controlling the vehicle has become automated, wherein automation would free up resources to visually attend to other areas of the driving environment.

Therefore, we propose this question: Do those with better “attentional function” distribute their visual attention more appropriately when driving? In other words, are those who may be better able to handle the attentional demands of driving also better able to distribute visual attention more appropriately? We use the term *attentional function* here to broadly describe an individual’s attentional control ability, that is, an ability to perform a number of attention tasks. It incorporates not only executive function abilities, for example, the ability to resolve cognitive conflict (Bush, Luu, & Posner, 2000), but also attention-alerting and attention-orienting aspects. These describe one’s level of attentional vigilance to impending stimuli and ability to select necessary information from various sensory inputs (Fan et al., 2009; Mackie, Van Dam, & Fan, 2013; Posner & Fan, 2008).

## Measuring Attentional Function and Its Relation to Driving Performance

Although little is known about the link between attentional function and eye movement behavior in driving, a number of studies have demonstrated links between attentional function and driving performance. For example, the useful field of view (UFOV) task (Ball, Roenker, & Bruni, 1990) aims to assess aspects of attention such as perceptual span, visual processing speed, and working memory function. Better performance on this task has been linked to better and, indeed, safer driving behavior (Ball, Owsley, & Beard, 1990; Ball, Owsley, Sloane, Roenker, & Bruni, 1993). Those who exhibit better attentional ability are therefore better able to handle the demands that driving places on the attentional systems. For example, they may be faster to respond to hazards or are better able to allocate attentional resources to the multitude of tasks involved when driving. We wish to explore if this observed better attentional control is also linked to a more effective pattern of eye movements when driving.

However, Bowers et al. (2011) noted that the UFOV task (and similar variations of this task) only measures selective and divided attention, it does not require sustained attention (attention over longer durations) to complete. In addition, the stimuli used are static. Sustained attention to dynamic stimuli is crucial to driving safely. One task that better captures these attentional aspects is the multiple-object tracking (MOT) task (e.g., Cavanagh & Alvarez, 2005). In a MOT task, observers are presented with a number of identical objects. Several of these are denoted as targets (usually by briefly increasing their visual salience by flashing) and the others are distractors. All objects will begin to move and the task is to maintain attention on all the target objects. At the end of the trial, the observer indicates which of the objects were the targets. Bowers et al. (2011) explored how performance on a MOT task relates to driving performance. Those who performed worse on the MOT task also had higher error scores on a road test (Bowers et al., 2011). In addition, MOT was found to be a stronger predictor than UFOV in predicting the ability to detect hazardous pedestrians during simulated driving in those with central visual field loss (Alberti, Horowitz, Bronstad, & Bowers, 2014). These results highlight not only the link between attentional function and driving but also suggest the importance of incorporating a dynamic assessment of sustained visual attention when studying driving performance. Therefore, in our study investigating the relationship between attentional function and eye movement behavior, we also utilized a MOT task to assess attentional function.

However, although MOT likely better captures the attentional properties required in driving more than tasks such as the UFOV, it does not include interactive visuomotor behavior. This may be important as the control of eye movements, attention and action can interact in a complex manner. For example, planning an action of either the eyes or the limbs can often facilitate the deployment of visual attention and information processing at the intended location (Hommel, 2010; Humphreys et al., 2010; Schneider & Deubel, 2002). Further, we often see the intrinsic temporal and spatial coupling between eye movements and action in everyday settings (Hayhoe & Ballard, 2005; Land, 2009; Land, Mennie, & Rusted, 1999; Tatler, Hayhoe, Land, & Ballard, 2011). In our previous work, we found that the way in which the oculomotor system is employed when actively engaged in a driving task was different than when passively viewing driving scenes (Mackenzie & Harris, 2015). Thus, we investigate how a more active visuomotor assessment of attention may correlate with eye movements and driving behavior.

With this requirement in mind, we drew on recent research which has investigated an interactive version of the MOT task (iMOT; Thornton, Bülthoff, Horowitz, Rynning, & Lee, 2014; Thornton & Horowitz, 2015). One of the aims of this work was to extend the standard MOT to capture more active attentional aspects of many everyday activities. In the iMOT task, the goal is to interact with the multiple objects on screen so that they avoid colliding with each other. So while individuals must divide their attention to multiple objects, they must also actively control hand movements to be successful in the task (see Thornton et al., 2014 for discussions).

In the current study, we use an altered version of this multiple-object avoidance (MOA) task to assess attentional function (in addition to the more typical MOT task mentioned above). In driving, although one must attend to multiple objects at once, only one object is being interacted with, namely, the car being driven. Therefore, we use a task we call a MOA. In this task, the goal is to control one object while avoiding multiple other objects (see Method section for details).

## Aims and Hypotheses

We have two aims in this study. The first aim is to identify if individuals who exhibit better attentional function also show better eye movement behavior when driving. We are taking an approach similar to that of “cognitive ethology” (Kingstone, Smilek, & Eastwood, 2008), where we wish to observe differences in eye movement behavior occurring naturally due to an individual’s own underlying cognitive processes. We explored the hypothesis in a group where all individuals had similar driving experience to each other, to identify links between individual differences in visual and driving performance and attentional function. Participants completed the two visual attention tasks described above (MOT and MOA) to measure attentional function and were then asked to drive a number of routes in a driving simulator program, while eye movements were tracked. Performance on the attention tasks was compared with visual behavior on the simulated driving task. We hypothesized that those who performed better in the attention tasks, thereby demonstrating better general attentional function, would exhibit more efficient visual behavior while driving.

For our second aim, we wished to extend previous research suggesting that dynamic and sustained attentional tasks provide useful predictors of driving behavior. Specifically, we compared the MOT and MOA tasks as predictors of driving performance and visual behavior. Although largely exploratory, we make the prediction that because the MOA task incorporates an active visuomotor component, it may predict the eye movement behavior involved in driving better than the more passively viewed MOT task.

## Method

### Participants

Twenty-seven participants took part in the study (12 males). Two participants were excluded due to poor eye movement calibration (>2°). This left a sample of 25 (11 males) with an age range of 18–51 years (*M*_age_ = 22.5 years; *SD* = 6.6). All participants had normal or corrected-to-normal vision and were recruited through the University of St. Andrews Sona Systems experiment participation scheme. They were paid £10 for participation. All participants had held a drivers’ license for at least 1 year (*M* = 4.3; *SD* = 5.7) and were from countries where driving on the left (e.g., United Kingdom) is standard. Participants reported having no previous experience with the driving simulator. Given the possible similarities between the driving simulation and the visual attentional tasks to a video game environment, we recruited participants who played video games, on average, less than 1 hr a week. The study was approved by the University of St Andrews University Teaching and Research Ethics Committee.

### Stimuli and Apparatus

**Figure 1 fig1:**
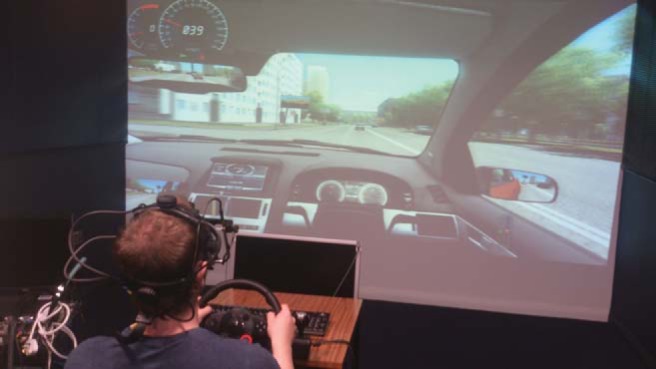
The basic experimental setup. Participants wore an eye tracker and were seated in front of a calibration screen and main projection screen. See the online article for the color version of this figure.

All testing was conducted at the University of St. Andrews’ Social Immersion suite. Participants performed both the driving simulation and attention tasks on the same viewing screen. Images were projected using an NEC MT1065 video projector (NEC Display Solutions, Tokyo, Japan). Participants sat 338 cm from the projection screen which had dimensions of 377 cm (58.3°) × 212 cm (34.8°; see Figure 1).[Fig-anchor fig1]

### Driving Simulation

**Figure 2 fig2:**
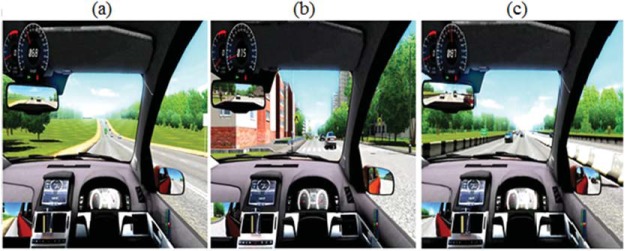
Screenshot images of the typical settings encountered in the (a) country highway, (b) urban area, and (c) motorway. Note the speedometer is located in the top left of the scene, with center rear-view mirror below it and passenger side mirror to the lower left. See the online article for the color version of this figure.

The driving simulator software used was City Car Drive (Forward Development, Moscow, Russia). With this software, we were able to program the properties of the car to mimic the feel of driving in the real world as closely as possible; including the vehicle’s inertia, brake torque, and mass. Side mirrors, a rear-view mirror, and speedometer were also available to the participants onscreen (see Figure 2 for instrument layout). The simulated field of view was 85°, similar to that in a real car. A Logitech Driving Force GT steering wheel and pedals combination was used to control the vehicle (Logitech, Lausanne, Switzerland). The virtual driving environments consisted of three courses, ordered by increasing complexity: (a) a country highway, (b) an urban driving scene, and (c) a motorway environment (see Figure 2). The country highway consisted of only single and dual lane carriageways with no chance of encountering pedestrians. The urban environment contained a number of extra potentially salient locations such as pedestrian crossings and contained sections with multiple lanes (up to three at times). Finally, the motorway consisted of fast moving traffic with multiple driving lanes and slip roads. Each contained a moderate level of traffic. The driving simulator software also tracked driving performance using a points system (see Measures section for more details).[Fig-anchor fig2]

### Visual Attentional Tasks

In order to assess attentional function, participants completed two visual attention tasks. Together, these tasks attempted to target a number of visual and attentional properties involved in driving (see introduction).

#### Multiple object tracking (MOT) task

**Figure 3 fig3:**
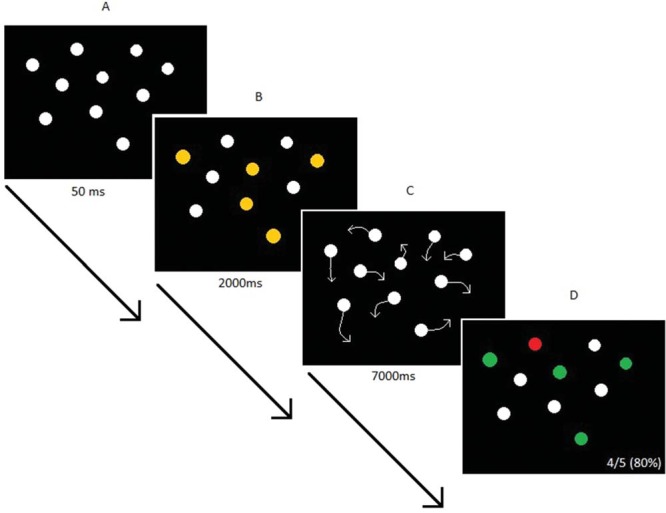
Multiple object tracking task. Participants are presented with the stimuli (A) briefly before five dots begin to flash orange (B). All dots turn back to white and then move randomly around the scene for seven seconds (C). Motion stops and the participant must select the five dots which had flashed orange (D). In this example, the participant has correctly identified four out of a possible five targets (The final positions of the dots would not be the same as the starting positions as pictured here—this is for illustrative purposes). See the online article for the color version of this figure.

The MOT task was programmed using EventIDE software (OkazoLab Ltd). Ten stationary white circles (diameter = 2.2°, luminance = 21.93 cd/m^2^) appeared on a black background on the screen (58.3° × 34.8°). After 50 ms, five flashed orange for 2 s. They returned to white and all 10 circles then moved around the display at random for 7 s. Motion speeds ranged from 4°/s to 9°/s and directions followed a random walk with constraints that circles did not overlap each other while moving. When the motion stopped, all 10 circles remained stationary until the participant indicated which five had originally flashed, by clicking on each with a mouse (see Figure 3). Immediate feedback was given to the participant indicating how many (out of five) had been correctly selected. The percent correct for each trial was taken as the performance measure, averaged across 30 trials. It is not appropriate to conduct correlations on this type of proportional data. Therefore, these scores were transformed using a Logit function, Ln[*p/*(1 − *p*)], where *p* is the percent correct performance score in the MOT across the 30 trials.[Fig-anchor fig3]

#### Multiple object avoidance (MOA) task

**Figure 4 fig4:**
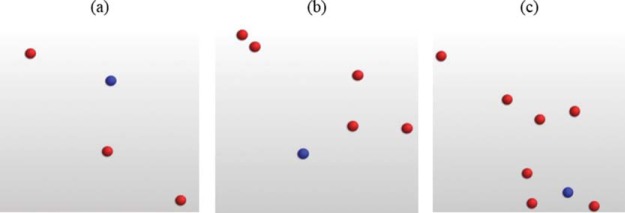
A static representation of the multiple-object avoidance task. The task starts with three red moving circles (a), then gets increasingly more difficult such as in (b) with five circles and in (c) with seven circles. See the online article for the color version of this figure.

Participants controlled a blue circle (diameter = 2.0°, luminance = 2.86 cd/m^2^) on the screen (size 34.5° × 32.2°) using the mouse. The task was to move the circle left, right, up, and down to avoid it touching a number of moving red circles (diameter = 2.0°, luminance = 2.86 cd/m^2^). Initially, three red circles were present. After 14 s, a new red circle appeared, and so on until the controlled blue circle collided with one of the red circles (see Figure 4). The total time (in seconds) of each trial was taken as a measure of performance (a longer time indicates better performance). Times were averaged across three trials. (Note that software for this task used was freely available online and was accessed by www.funnygames.co.uk/avoid-the-balls.htm. It was not programmed by the experimenters, and, therefore, specific parameters of the task, e.g., circle movement speed, could not be altered.)[Fig-anchor fig4]

### Eye Movement Recording

An SR Research Eyelink II head-mounted eye tracking system was used to record eye movements, sampling binocularly at 250 Hz. Fixations and saccades were determined using a displacement threshold of 0.1°, a velocity threshold of 30°/s and an acceleration threshold of 8,000°/s^2^ (SR Research Ltd, Ottawa, Canada). An initial 12-point screen calibration using a secondary screen at a distance of 98 cm was performed to ensure that recordings had a mean spatial error of less than 0.5°. This screen was lowered away from the field of view during recording. A 9-point depth calibration was conducted on the stimulus display screen at a distance of 338 cm to correct for depth parallax. Participants were free to move their head.

### Measures

#### Eye movement measures

**Figure 5 fig5:**
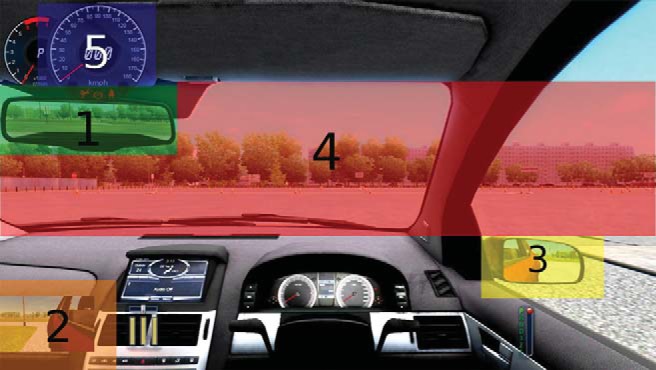
Illustration of each area of interest. (1) Rear-view mirror (16° × 5°), (2) passenger-side mirror (12° × 5°), (3) driver-side mirror (19° × 7°), (4) roadway (58° × 27° at maximum length and height), (5) speedometer (12° × 9°). See the online article for the color version of this figure.

All eye movement information was recorded and collated via SR Research Data Viewer software. Using this software, the driving scene was divided into five different interest areas (see Figure 5): the rear-view mirror, driver-side mirror, passenger-side mirror, speedometer and the roadway. The passenger-side mirror was superimposed on the bottom-left of the screen and the speedometer was superimposed on the top left of the screen.[Fig-anchor fig5]

##### Fixation locations/spread of visual attention

The standard deviations of eye fixation locations along the horizontal axis (using *x*-axis pixel coordinates) were measured to provide an indicator of the spread of visual attention (e.g. Chapman & Underwood, 1998). A larger standard deviation would suggest a larger distribution of fixations and thus a greater spread of visual attention. Only fixations located within the roadway were included in this analysis; mirror or speedometer fixations were excluded.

##### Mirror and speedometer interest area analyses

To measure how much individuals inspected the vehicle mirrors and the speedometer, the average fixation dwell time (as a percentage of the total drive time) was calculated for the rear-view mirror, driver-side mirror, passenger-side mirror, and the speedometer.

##### Saccade information

We recorded the average saccade velocities to infer the efficiency at which the scene was sampled, where faster average saccades corresponds to increased information processing. We also recorded the average size of the saccades and the number of saccades made. We performed saccade analyses for the overall scene (i.e., all interest areas) and for the roadway interest area separately.

#### Driving performance

Driving performance was evaluated using a demerit-based point system, similar to methods used to measure driving ability (e.g., Bowers et al., 2011; Weaver, Bédard, McAuliffe, & Parkkari, 2009) and to standard on-road tests such as the U.K. driving test. Demerit points were awarded for infractions in four categories of driving safety: (a) general control of the vehicle/maneuvers (e.g., lane positioning, turning and overtaking), (b) attending to priority (right of way), (c) signal violations, and (d) speed violations. Within these categories, either 500 or 1,000 points were awarded depending on the severity of the infraction. As examples, a minor infraction (500 points) would be awarded for crossing the lane markers, and a major infraction (1,000 points) would be awarded for causing another vehicle to unexpectedly brake hard. The points were awarded and tracked by the driving software, not by the experimenter. The total demerit points awarded provided a measure of driving performance where a larger number of points would suggest poorer performance. It was not possible to isolate the driving performance score for each course individually, therefore the driving performance score was a measure across the entire driving session. Participants were not told that their driving performance would be measured.

### Procedures

All participants completed a two-part study on driving and visual attention, one part being the driving simulation and the other being the visual attention tasks. All participants first completed a questionnaire examining their level of vision and driving experience. Potential participants completed a Landolt C visual acuity test and were included if acuity was measured as <2.0 minimal angle resolution. Thirteen participants performed the driving task first and 12 participants performed the attention tasks first. Breaks were given between tasks and at any point required by the participant.

For the driving task, participants were presented with the first person viewpoint of a car in a large car park on screen. They were instructed in how to use the car, including how to steer, use the pedals and turn signals. They were also informed about the location of the vehicle mirrors. They were then given 5 min to practice the simulated driving in the car park and informed they would be completing a number of set routes. Eye movements were calibrated using the Eyelink II at both the calibration distance and at the video screen distance. Calibration was done before each course and recording began at the start of each course just as participants began to drive. The order of driving the three courses was randomized. For the country highway, participants were instructed to follow the road at the beginning of the drive. For the motorway course, participants were instructed to follow the motorway until a certain exit was to be taken. For the urban district, participants were instructed to take three turns (a left turn, a right turn, and another left turn) at certain points on the course. Instructions were given by the experimenter at least 10 s in advance of the turn to avoid awkward or dangerous maneuvering of the vehicle by the participant. After a certain location was reached (known only to the experimenter) in each of the courses, recording of the eye movements stopped, and the participant was instructed to stop the vehicle.

The order of the attention tasks was completed based on a Latin square design to guard against practice effects. Although not relevant for the purposes of this current study, eye movements were calibrated and tracked for each of the tasks. For the MOT task, participants were instructed to maintain attention to five circles on screen from a total of 10. They were told to pay attention to the five circles that flashed orange at the beginning of each trial and try to maintain attention on these circles as they moved around the screen. At the end of the trial, they used a mouse to identify which circles had flashed orange. Five practice trials were given before they completed all 30 experimental trials.

For the MOA, participants were instructed to control the blue circle on screen with the mouse and had to actively avoid the moving red circles. They were informed that more red circles would continue to appear as the trial went on. One practice trial was given before three experimental trials were completed. Each trial ended when the blue circle touched one of the red circles. The complete experiment lasted a maximum of 2 hr.

### Statistical Design

Pearson correlations were used to identify the relationship between performance in the two attention tasks and each of the measures described above. Multiple linear regression analyses were also conducted for the measures which showed strong relationships with attentional function. This allowed us to investigate how well each task predicts driving performance and eye movement behavior. Driving experience was considered as a covariate in the analyses. However, because driving experience was kept similar across participants, it did not correlate with any of the measures. It was therefore not entered into the analyses. A power calculation was conducted investigating the sample size needed to obtain a power of 0.8, when correlative effect size is moderate to strong (*R*^2^ = 0.25). The calculation established that a sample of 23 was required (Faul, Erdfelder, Buchner, & Lang, 2009).

## Results

For the MOA task, performance was measured as the time (in seconds) until the target object collided with any of the other objects. This was averaged across three trials. For the MOT task, performance was measured as the percentage number of correct targets selected out of five. This was averaged across 30 trials. Descriptive statistics for performance in the two attention tasks are given in Table 1. To investigate the relationship between the two visual attention tasks, a Pearson correlation was conducted. As it is not appropriate to conduct correlations on proportional data, the MOT scores were transformed using a Logit function: Ln[*p*/(1 − *p*)], where *p* is the proportion correct MOT score. Performance between these two tasks was strongly positively correlated, *r*(25) = 0.6, *p* = .004. This is unsurprising given that these tasks aim to target similar attentional tracking ability.[Table-anchor tbl1]

Using performance in these two tasks as predictors, in the next sections we report correlations and regressions to (a) investigate the relationship between attentional function and driving performance/eye movement behavior and (b) to examine how well each of these tasks predict the driving measures.

### Driving Performance and Horizontal Scanning Behavior

**Figure 6 fig6:**
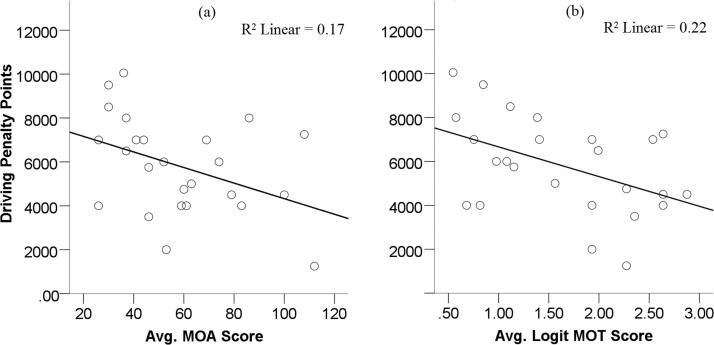
Relationship between performance in the attention tasks and driving performance. (a) Multiple object avoidance task (MOA); (b) multiple-object tracking task (MOT).

Driving performance data were recorded for the overall drive, not for each individual course, by the software. Figure 6 shows the relationship between performance on each of the two attention tasks and driving performance. It is clear that those who performed better in the attention tasks obtained less driving penalty points. Pearson correlations showed that this relationship was significant for the MOA, *r*(25) = −0.41, *p* = .044 (Figure 6a), and the MOT, *r*(25) = −0.47, *p* = .018 (Figure 6b). These results suggest that those with better attentional function performed better in the simulated drive.[Fig-anchor fig6]

Data were entered into a regression to explore these results further. A hierarchical regression was used, entering the MOT task into the model first. We were interested in examining how much more variation in driving performance could be explained by adding the MOA data into the model. The first model (only MOT) significantly predicts driving performance, *F*(1, 23) = 6.39, *p* = .019, *R*^2^ = 0.22. When MOA performance was included in the model, the change in *R*^2^ was 0.031, and this change was not significant, *F* = 0.91, *p* = .35. The overall model remained significant, *F*(2, 22) = 3.63, *p* = .043, *R*^2^ = 0.25; MOT coefficients: *b* = −746.27, β = −.35, *t* = −1.56; MOA coefficients: *b* = −18.51, β = −0.21, *t* = −0.95. These results suggest that both tasks predict driving performance; however, they share a very similar proportion of the variation in explaining driving performance.

**Figure 7 fig7:**
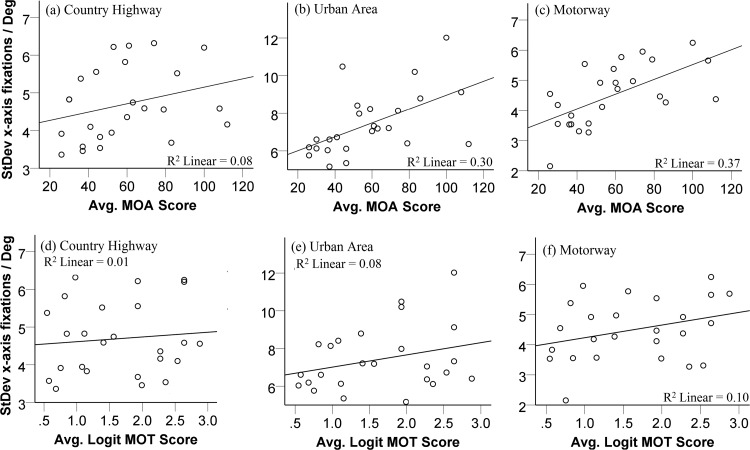
Correlations between performance on the attention tasks and horizontal scanning behavior, as measured by the standard deviation of *x*-axis fixations, for each of the three courses, Country Highway, Urban Area, and Motorway. (a–c) Multiple-object avoidance task (MOA); (d–f) multiple-object tracking task (MOT).

Figure 7 shows the relationship between performance in the two attention tasks and horizontal scanning behavior. Unlike our measure for driving performance, we were able to measure eye movements separately for each of the three courses. Pearson correlations were conducted. For the country highway route, there was no clear relationship between attentional function and horizontal scanning behavior, MOA: *r*(25) = 0.29, *p* = .16; MOT: *r*(25) = 0.10, *p* = .32 (Figures 7a and 7d, respectively). However, for the more complex routes, performance on the MOA significantly positively correlated with a wider horizontal scan, urban area: *r*(25) = 0.55, *p* = .004, and motorway: *r*(25) = 0.61, *p* = .001 (Figures 7b and 7c, respectively). Performance on the MOT showed a weak relationship and was not significantly correlated with a wider scan for either of these courses, urban area: *r*(25) = 0.29, *p* = .16, and motorway: *r*(25) = 0.31, *p* = .13 (Figures 7e and 7f, respectively). These results suggest that better attentional function, as measured only by the MOA, is related to exhibiting a wider visual search during the more complex driving routes.[Fig-anchor fig7]

Data for the urban area and motorway were entered into a multiple regression model to obtain predictor coefficients. Because MOT did not significantly correlate with horizontal scanning behavior, only the MOA was entered as an individual predictor of horizontal scanning, urban area: *b* = 0.51, β = 0.55, *t* = 3.15, *p* = .004; motorway: *b* = 0.34, β = 0.61, *t* = 3.68, *p* = .001. These analyses suggest that the MOA is a moderate predictor of horizontal scanning behavior.

### Area of Interest Fixation Dwell Times (Mirror and Speedometer Use)

For each of the three courses, correlations were conducted between performance in the attention tasks and the time spent fixating in the three vehicle mirrors (as measured in percentage fixation dwell times). These can be viewed in Table 2.[Table-anchor tbl2]

From Table 2 it is clear that there is not a strong relationship between attentional function, as measured by the attention tasks, and overall time spent fixating the mirrors. There was however a significant positive correlation between task performance and the time spent fixating the passenger side mirror during the country highway course, MOA: *r*(25) = 0.58, *p* = .002; MOT: *r*(25) = 0.40, *p* = .049, highlighting that those with better attentional function spent more time fixating in this mirror. No multiple regression models were considered here given the general pattern of results.

**Figure 8 fig8:**
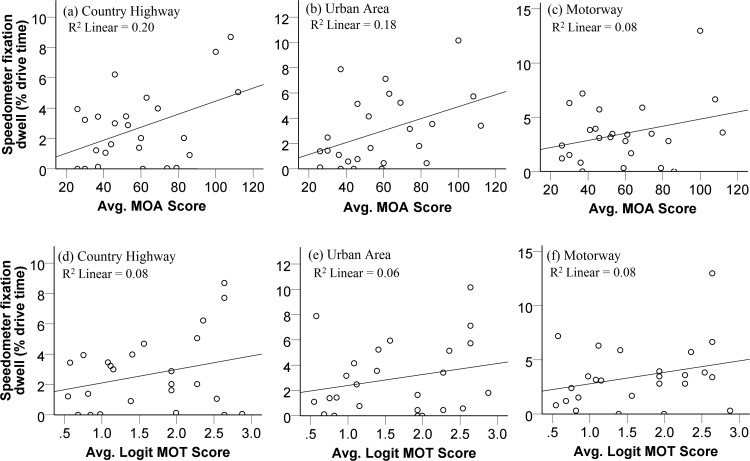
Correlations between performance in the attention tasks and use of the speedometer, as measured by the percentage fixation dwell time, for each course.

Figure 8 shows the relationship between performance on the attention tasks and the time spent fixating the speedometer in each course. Performance on the MOA significantly positively correlated with the time spent fixating the speedometer during the country highway drive, *r*(25) = 0.45, *p* = .036 (Figure 8a), and urban drive, *r*(25) = 0.42, *p* = .035 (Figure 8b), but not when driving on the motorway, *r*(25) = 0.29, *p* = .17 (Figure 8c). Performance on the MOT task did not significantly correlate with the time spent fixating the speedometer during any of the drives, country highway: *r*(25) = 0.28, *p* = .18; urban area: *r*(25) = 0.23, *p* = .26; motorway: *r*(25) = 0.29, *p* = .18 (Figures 8d, 8e, and 8f, respectively). Together, these results suggest that those with better attentional function, as measured only by the MOA, fixated their speedometers more during most of the drives.[Fig-anchor fig8]

Data for the country highway and urban area were entered into a multiple regression model to obtain predictor coefficients. Because MOT did not significantly correlate with speedometer use, only the MOA was entered as an individual predictor of speedometer use, country highway: *b* = 0, β = 0.45, *t* = 2.39, *p* = .026; urban area: *b* = 0, β = 0.42, *t* = 2.24, *p* = .035.

### Saccadic Eye Movements

For this analysis, we were interested in the relationship between attentional function and the velocity of saccades and the size of saccades made. We separated the saccades that were made when inspecting the roadway area of interest and those inspecting the overall scene. To be concise, data were averaged across the three courses to give a general view of saccadic patterns. Individual Pearson correlations can be viewed in Table 3.[Table-anchor tbl3]

Performance in the MOA significantly positively correlated with the velocity of saccades made within the roadway, *r*(25) = 0.44, *p* = .029, and the overall scene, *r*(25) = 0.52, *p* = .007. MOA performance also significantly positively correlated with the size of saccades made within the roadway, *r*(25) = 0.43, *p* = .031, and the overall scene, *r*(25) = 0.42, *p* = .036. These results suggest those with better attentional function, as measure by the MOA, exhibited faster and larger saccades when driving. Importantly, this was independent of the number of saccades made, where there was no relationship between MOA performance and the number of saccades made during inspection of the roadway, *r*(25) = −.011, *p* = .6, and the overall scene, *r*(25) = −0.01, *p* = .98. Performance on the MOT task did not significantly correlate with saccade behavior (see Table 3).

To obtain predictor coefficients, data were entered into multiple regression models. Only MOA was entered as a sole predictor of saccade behavior because MOT did not correlate: roadway saccade velocity (*b* = 0.3, β = 0.44, *t* = 2.32, *p* = .029); overall saccade velocity (*b* = 0.56, β = 0.52, *t* = 2.95, *p* = .007); roadway saccade size (*b* = 0.01, β = 0.43, *t* = 0.29, *p* = .031); overall saccade size (*b* = 0.03, β = 0.42, *t* = 2.22, *p* = .036).

## Discussion

The first aim of this study was to use a specific set of visual attention tasks to test whether individual differences in eye movement behavior when driving may be partly due to one’s ability to manage attentional demands. We made the specific hypothesis that those individuals who performed better on the attention tasks, and thus have better attentional function, would exhibit more effective visual and driving behavior. We found a number of results that support this, and below, we discuss these in the context of the existing literature. The second aim was to investigate how well each of the two tasks (MOT and MOA) predicts driving eye movement behavior and driving performance. These aims are discussed separately.

### Attentional Function and Driving Ability

We found that better attentional function is related to better overall driving performance, which is in line with many other studies highlighting the relationship between attentional function and driving performance (Aksan, Anderson, Dawson, Uc, & Rizzo, 2015; Anstey, Horswill, Wood, & Hatherly, 2012; Keay et al., 2009; Roca, Crundall, Moreno-Ríos, Castro, & Lupiáñez, 2013; Weaver et al., 2009). Driving is a demanding attentional task and a better driver is likely one who can, for example, successfully attend to relevant areas while ignoring other stimuli, orient their attention to potential hazardous cues, and sustain attention to the dynamic driving environment. This perhaps helps to explain why those who perform better in attention tasks also exhibit better, or indeed, safer, driving behavior.

### Attentional Function and Eye Movements

Competition for attentional resources during driving may limit scanning behavior (Engström et al., 2005; Recarte & Nunes, 2003; Savage et al., 2013). Thus, the level of cognitive load experienced by a driver may be a likely source for individual differences in drivers’ eye movements. Although related to this idea, in this study, we did not manipulate levels of cognitive load when driving. Instead we measured attentional function in a separate series of tasks. We found evidence that those with better attentional function exhibit more effective eye movement behavior (measured mainly by the MOA as discussed in a later section). Importantly, this is eye movement behavior we would typically associate with more experienced or safer drivers (Crundall et al., 2003; Konstantopoulos et al., 2010). The evidence here suggests that those who have better control over attention resources are better able to distribute eye movements to more relevant areas of the driving scene, as shown by increased horizontal scanning (see Figure 7).

This is evidenced further by the finding that the effect appears to become more pronounced when road complexity increases. Previous research has found differences in eye movement strategies due to the different processing demands of the road type (Chapman & Underwood, 1998; Crundall & Underwood, 1998; Underwood, Chapman, et al., 2002). For example, Crundall and Underwood (1998) showed that the size of horizontal visual scanning on the roadway was similar for novices and experienced drivers on rural and suburban routes. However, on dual carriageways, where the layout is much more complex (e.g., presence of slip roads), only experienced drivers exhibited a wider horizontal visual scanning strategy. Similarly, in this study, for the less demanding country highway, there was no relationship between attentional function and horizontal scanning behavior. It could be that the lower demands of the route allow individuals to successfully distribute eye movements across the scene. When the scene became increasingly complex, that is, in urban or motorway environments, we found a significant correlation between increased scanning behavior in those with better attentional function. The more complex driving environments may place a higher cognitive load on the visual and attentional systems that could limit scanning behavior in those with poorer attentional function.

These findings suggest that those with better attentional function may be better equipped to search the road more for hazards. Inattention and failures to scan the roadway are often contributing factors to road accidents (Dingus et al., 2006; Klauer et al., 2006; Lee, 2008; Lestina & Miller, 1994). Thus, our findings may suggest that the reasons for these contributing factors are due to poor attentional function.

Much like scanning the roadway, some research has suggested that increasing cognitive load reduces mirror use (Harbluk, Noy, Trbovich, & Eizenman, 2007; Recarte & Nunes, 2003). Given our finding that those with better attentional function were better able to deploy eye movements across the roadway, one might predict (as we did) that they would be better equipped to increase inspection of the mirrors—a desirable behavior exhibited more often in experienced drivers (Konstantopoulos et al., 2010; Underwood, Crundall, & Chapman, 2002). However, the results did not support this. Only fixations pertaining to the passenger-side mirror on the country highway course showed this relationship, suggesting that inspection of the mirrors cannot easily be explained by an individual’s attentional function—at least as measured by our tasks. These findings might not be too surprising. Even if an individual has poorer attentional function, vehicle mirrors, particularly the rear-view mirror and driver-side mirror, are still hugely important when driving. They provide the driver with added information about the surroundings and the necessary safety information with which to make informed decisions about making maneuvers—particularly, for example, when attempting to overtake other road users. Thus their more immediate importance to safety may mean that all drivers invest cognitive effort in using them.

It is interesting to find that, in this study, those with better attentional function spent more time inspecting the speedometer during the country highway and urban drives (see Figure 8). This suggests that these individuals are better able to allocate visual attention resources to monitor vehicle speed more often. This may have important implications for driver safety, with speeding being one of the most commonly reported reasons for road accidents (Cooper, 1997; Mesken, Lajunen, & Summala, 2002). There are a number of explanations given as to why individuals speed, for example, attitude (Elliott, Lee, Robertson, & Innes, 2015), and our finding here may suggest that one other reason is that individuals simply fail to monitor their speed as often because attention is allocated to other aspects of driving. Although, one should consider that this was simulated driving and therefore the consequences for not monitoring vehicle speed is reduced considerably. In addition, compared to the position of a speedometer in a real car, the effort required to inspect the speedometer here is likely more, given its position in the top left portion of the viewing screen. This may have exaggerated the relationship between attentional function and inspection time. Inspection of the speedometer here may not reflect inspection on real roads therefore.

We find some evidence to suggest individuals with better attentional function are more efficient at visually sampling the scene as evidenced by the average faster saccade velocities (see Table 3). Mean saccade velocity has previously been used to infer information processing, where faster saccades have been associated with increased information processing (Galley & Andres, 1996) and the converse, where smaller velocities are associated with lower levels of vigilance (Galley, 1989, 1993). We therefore propose our finding may be an indicator of increased processing performance for those with better attentional function. It could also be argued that this increase in eye movement velocity was simply a product of those with better attentional function making larger saccades (see Table 3). This might be true, but given the high correlation between saccade velocity and amplitude (Baloh, Sills, Kumley, & Honrubia, 1975; Schmidt, Abel, Dell’Osso, & Daroff, 1979), it would be hard to tease apart these factors with the data here. What we argue is important is that the velocity and amplitude of saccades were independent of the number of saccades that were made. This suggests that those with better attentional function were better able to distribute eye movements around the driving scene and this was not at a cost of making more eye movements.

### Comparison With Studies Comparing Experienced and Novice Driving

The current study investigated individual differences in eye movements in a population with similar driving experience. However, we can draw parallel conclusions with the literature concerning the differences in eye movement behavior between novice and experienced drivers. We provide support for the idea that visual scanning differences may be due to the competing attentional resources required to both drive the vehicle appropriately and observe the roadway for potential hazards (Crundall & Underwood, 1998; Underwood, Chapman, et al., 2002). For novices, driving is not a highly practiced task (in comparison to the many years of practice that experienced drivers possess), thus more resources may be required for vehicle control. Novices, for example, might prioritize fixating on points on the road which aid steering, for example, “future path” points (Kountouriotis, Floyd, Gardner, Merat, & Wilkie, 2012; Lappi, 2014) or fixate closer to the vehicle to maintain lane position (Mourant & Rockwell, 1972). We know that through practice and experience, task performance improves when actions become more automated and there is less of a requirement for conscious intervention (Ackerman, 1988; Moors & De Houwer, 2006). With driving, it may be the case that through experience, fewer conscious resources are required for controlling the vehicle as driving skill becomes automatic and this frees up resources to allocate visual attention to other parts of the scene.

This may also explain the individual differences we observe here: controlling the vehicle may require more attentional resources in some individuals, resulting in less attentional resources remaining for scanning the road. These results suggest that some individuals may be better equipped for predicting, detecting and responding to hazards. Even if an individual has the knowledge of where to look, if fewer attentional resources limit their ability to scan certain areas of the roadway, then this in turn may limit their hazard perception ability.

### A Place for Visuomotor Assessment Tools?

Bowers et al. (2011) discuss how the UFOV, and similar tasks, only measures selective and divided attention. It does not require sustained attention to complete. In UFOV, stimuli are only presented for up to several hundred milliseconds and, thus, only capture brief spans in attention. Driving is a more complex task, and the attentional mechanisms involved in driving may not be accurately represented when performing the UFOV task. The MOT, which is a more dynamic and sustained assessment of executive control, was proposed and was found to correlate to driving performance (Alberti, Horowitz, et al., 2014; Bowers et al., 2011). In this current study, we also found evidence to support the claims that better performance on the MOT predicts better driving performance (see Figure 6).

However, one of our aims was to provide further insights into the types of tasks which can be used to predict overall driving behavior by investigating tasks which incorporate visuomotor control. Our MOA task was based on the iMOT (Thornton et al., 2014). While also requiring the sustained attentional aspect to dynamic stimuli, the objective was to actively control one object to avoid the multiple other objects that would appear. With this, we aimed to capture the intrinsic link between vision and action seen in many common everyday tasks (Land, 2006; Land & Tatler, 2009; Steinman, 2003). Thus, we hypothesized that performance in this task would better predict the active eye movement behavior in driving more than the MOT. The results confirmed this in most of our eye movement measures.

A MOT-type task is passive in nature which does not require active visuomotor control. The eye movement strategies involved are likely different to a more active task, one which incorporates the vision and action link we see in many everyday tasks (Hayhoe & Ballard, 2005; Land et al., 1999). In this case, the MOA task requires vision to initially select a point in space in which to move the ball to, which precedes the action of moving the ball. In this task, many eye movements are required to be successful in the task. We know that a visual strategy often used in MOT is to make fewer eye movements and use covert attention to group stimuli (Fehd & Seiffert, 2008; Oksama & Hyönä, 2016; Zelinsky & Neider, 2008). Indeed, we found that individuals made significantly fewer fixations in the MOT task than the MOA task (MOT mean fixations per second: 2.3, MOA mean fixations per second: 2.7), *t*(20) = 3.1, *p* = .006. This may explain why the MOT task does not significantly predict eye movement scanning behavior in a more active task such as driving, where eye movements should ideally be deployed to many parts of the environment.

Both the MOT and MOA did predict driving performance. We would therefore suggest both tasks are useful when investigating attentionally complex tasks, such as driving. In this experiment, they shared a similar proportion of the variation in explaining driving performance scores, with the MOT performance explaining marginally more. One could argue that, ultimately, predicting driving performance is the more important factor than predicting it along with eye movements. We agree with this argument to a certain extent, if one assumes that more effective eye movements is simply a contributor to overall driving performance. For example, scanning the road more for potential hazards may allow an individual to identify them and therefore respond earlier if the hazard develops. However, in the current experiment, the driving scenes were not hazardous, where only a moderate level of traffic was simulated throughout and the other road users were not programmed to be aggressive. The increased scanning of the road observed for those with better attention performance would not necessarily have directly translated into better driving performance given the traffic conditions. Thus, this direct link between eye movements and driving performance cannot be easily identified with the current data. It would be interesting to investigate how these tasks predict performance in more hazardous or demanding road situations.

One limitation to note is that we have not directly compared how well the MOA (or MOT) predicts driving behavior relative to the more standard tasks used for example, UFOV. This would need to be done to answer a more explicit question: Which task is the most useful predictor tool for driving and visual behavior? This highlights a potential follow up to this research.

## Conclusions

We have found that there are individual differences in eye movement behavior and driving performance even among those with similar driving experience. We found that individual’s attentional function is a contributing factor to these differences; where better performance on visual attention tasks is accompanied by eye movement and driving behavior typically associated with safer driving. We showed this without explicitly inducing a high cognitive demand during driving, to maintain a more naturalistic driving setting. We also provided evidence to suggest that tasks utilizing a visuomotor component may provide useful prediction tools for driving and eye movements together. Our results thus provide new insights into how the visual and attentional systems interact during everyday tasks.

## Figures and Tables

**Table 1 tbl1:** Descriptive Statistics for the Multiple-Object Avoidance Task (MOA) and the Multiple-Object Tracking Task (MOT)

Attention task	*N*	Minimum	Maximum	*M*	*SD*
MOA	25	26 s	112 s	58.32 s	25.22 s
MOT	25	63%	97%	81%	10.62%
*Note.* s = Seconds.					

**Table 2 tbl2:** Correlations Showing the Relationship Between Performance in the Attention Tasks and the Time Spent Fixating the Vehicle Mirrors, as Measured by the Percentage Time Spent Fixating, for Each Course

Attention task and statistic	Country highway			Urban area			Motorway		
	Rear	Driver	Pass	Rear	Driver	Pass	Rear	Driver	Pass
MOA									
*r*	.26	−.03	.58**	.08	−.17	−.12	.08	−.35	.18
*p*	.21	.9	.002	.71	.41	.59	.72	.09	.40
MOT									
*r*	−.12	.33	.40*	.04	−.25	.04	.08	.01	.36
*p*	.58	.12	.049	.86	.24	.087	.7	.96	.07
*M*	2.8	2.8	.6	3.0	2.9	1.3	3.6	4.4	1.3
*SD*	2.8	3.5	.6	2.3	3.9	1.1	3.1	3.2	1.3
*Note*. Rear = rear-view mirror; Driver = driver-side mirror; Pass = passenger-side mirror; MOA = Multiple-object avoidance task; MOT = Multiple-object tracking task.									
* Significance at *p* = .05 level. ** Significance at *p* = .01 level.									

**Table 3 tbl3:** Correlations Showing the Relationship Between Performance in the Attention Tasks, Roadway Saccade Behavior, and Overall Saccade Behavior

Attention task and statistic	Saccade velocity per degrees per second		Saccade size per degree		Saccades per second	
	Roadway	Overall	Roadway	Overall	Roadway	Overall
MOA						
*r*	.44*	.52*	.43*	.42*	−.11	−.01
*p*	.029	.007	.031	.036	.60	.98
MOT						
*r*	.18	.21	.04	.047	.19	.07
*p*	.4	.31	.86	.824	.37	.74
*M*	86.6	114.95	2.90	5.81	1.79	2.66
*SD*	17.27	27.12	.63	1.75	.47	.70
*Note*. MOA = multiple-object avoidance; MOT = multiple-object tracking.						
* Significance at *p* = .05 level.						

## References

[c1] Ackerman P. L. (1988). Determinants of individual differences during skill acquisition: Cognitive abilities and information processing. Journal of Experimental Psychology: General, 117, 288–318. 10.1037/0096-3445.117.3.288

[c2] Aksan N., Anderson S. W., Dawson J., Uc E., & Rizzo M. (2015). Cognitive functioning differentially predicts different dimensions of older drivers’ on-road safety. Accident Analysis and Prevention, 75, 236–244. 10.1016/j.aap.2014.12.007 25525974 PMC4386614

[c3] Alberti C. F., Horowitz T., Bronstad P. M., & Bowers A. R. (2014). Visual attention measures predict pedestrian detection in central field loss: A pilot study. PLoS ONE, 9, e89381 10.1371/journal.pone.0089381 24558495 PMC3928437

[c4] Alberti C. F., Shahar A., & Crundall D. (2014). Are experienced drivers more likely than novice drivers to benefit from driving simulations with a wide field of view? Transportation Research Part F: Traffic Psychology and Behaviour, 27, 124–132. 10.1016/j.trf.2014.09.011

[c5] Anstey K. J., Horswill M. S., Wood J. M., & Hatherly C. (2012). The role of cognitive and visual abilities as predictors in the multifactorial model of driving safety. Accident Analysis and Prevention, 45, 766–774. 10.1016/j.aap.2011.10.006 22269568

[c6] Ball K., Owsley C., & Beard B. (1990). Clinical visual perimetry underestimates peripheral field problems in older adults. Clinical Vision Sciences, 5, 113–125.

[c7] Ball K., Owsley C., Sloane M. E., Roenker D. L., & Bruni J. R. (1993). Visual attention problems as a predictor of vehicle crashes in older drivers. Investigative Ophthalmology & Visual Science, 34, 3110–3123. 8407219

[c8] Ball K., Roenker D. L., & Bruni J. R. (1990). Developmental changes in attention and visual search throughout adulthood. Advances in Psychology, 69, 489–508. 10.1016/S0166-4115(08)60472-0

[c9] Baloh R. W., Sills A. W., Kumley W. E., & Honrubia V. (1975). Quantitative measurement of saccade amplitude, duration, and velocity. Neurology, 25, 1065–1070. 10.1212/WNL.25.11.1065 1237825

[c10] Bowers A., Anastasio J., Howe P., O’Connor M., Hollis A., Kapust L., . . . Horowitz T. (2011). Dynamic attention as a predictor of driving performance in clinical populations: Preliminary results. Paper presented at the Proceedings of the Sixth International Driving Symposium on Human Factors in Driver Assessment, Training, and Vehicle Design. Lake Tahoe, CA.

[c11] Bush G., Luu P., & Posner M. I. (2000). Cognitive and emotional influences in anterior cingulate cortex. Trends in Cognitive Sciences, 4, 215–222. 10.1016/S1364-6613(00)01483-2 10827444

[c12] Cavanagh P., & Alvarez G. A. (2005). Tracking multiple targets with multifocal attention. Trends in Cognitive Sciences, 9, 349–354. 10.1016/j.tics.2005.05.009 15953754

[c13] Chapman P. R., & Underwood G. (1998). Visual search of driving situations: Danger and experience. Perception, 27, 951–964. 10.1068/p270951 10209634

[c14] Cooper P. J. (1997). The relationship between speeding behaviour (as measured by violation convictions) and crash involvement. Journal of Safety Research, 28, 83–95. 10.1016/S0022-4375(96)00040-0

[c15] Corbetta M., Akbudak E., Conturo T. E., Snyder A. Z., Ollinger J. M., Drury H. A., . . . Shulman G. L. (1998). A common network of functional areas for attention and eye movements. Neuron, 21, 761–773. 10.1016/S0896-6273(00)80593-0 9808463

[c16] Crundall D., Chapman P., Phelps N., & Underwood G. (2003). Eye movements and hazard perception in police pursuit and emergency response driving. Journal of Experimental Psychology: Applied, 9, 163–174. 10.1037/1076-898X.9.3.163 14570510

[c17] Crundall D., & Underwood G. (1998). Effects of experience and processing demands on visual information acquisition in drivers. Ergonomics, 41, 448–458. 10.1080/001401398186937

[c18] Dingus T. A., Klauer S., Neale V., Petersen A., Lee S., Sudweeks J., . . . Gupta S. (2006). The 100-car naturalistic driving study, Phase II-results of the 100-car field experiment (National Highway and Traffic Safety Administration Report No. DOT HS 810 593). Washington, DC: National Highway and Traffic Safety Administration.

[c19] Elliott M. A., Lee E., Robertson J. S., & Innes R. (2015). Evidence that attitude accessibility augments the relationship between speeding attitudes and speeding behavior: A test of the MODE model in the context of driving. Accident Analysis and Prevention, 74, 49–59. 10.1016/j.aap.2014.10.007 25463944

[c20] Endsley M. R. (1995a). Measurement of situation awareness in dynamic systems. Human Factors: The Journal of the Human Factors and Ergonomics Society, 37, 65–84. 10.1518/001872095779049499

[c21] Endsley M. R. (1995b). Toward a theory of situation awareness in dynamic systems. Human Factors: The Journal of the Human Factors and Ergonomics Society, 37, 32–64. 10.1518/001872095779049543

[c22] Endsley M. R. (2004). Situation awareness: Progress and directions. A cognitive approach to situation awareness: Theory, measurement and application, 317–341.

[c23] Endsley M. R., & Garland D. J. (2000). Situation awareness analysis and measurement. Mahwah, NJ/London, UK: Lawrence Erlbaum Associates.

[c24] Engström J., Johansson E., & Östlund J. (2005). Effects of visual and cognitive load in real and simulated motorway driving. Transportation Research Part F: Traffic Psychology and Behaviour, 8, 97–120. 10.1016/j.trf.2005.04.012

[c25] Falkmer T., & Gregersen N. P. (2001). Fixation patterns of learner drivers with and without cerebral palsy (CP) when driving in real traffic environments. Transportation Research Part F: Traffic Psychology and Behaviour, 4, 171–185. 10.1016/S1369-8478(01)00021-3

[c26] Fan J., Gu X., Guise K. G., Liu X., Fossella J., Wang H., & Posner M. I. (2009). Testing the behavioral interaction and integration of attentional networks. Brain and Cognition, 70, 209–220. 10.1016/j.bandc.2009.02.002 19269079 PMC2674119

[c27] Faul F., Erdfelder E., Buchner A., & Lang A.-G. (2009). Statistical power analyses using G*Power 3.1: Tests for correlation and regression analyses. Behavior Research Methods, 41, 1149–1160. 10.3758/BRM.41.4.1149 19897823

[c28] Fehd H. M., & Seiffert A. E. (2008). Eye movements during multiple object tracking: Where do participants look? Cognition, 108, 201–209. 10.1016/j.cognition.2007.11.008 18096148 PMC2430980

[c29] Galley N. (1989). Saccadic eye movement velocity as an indicator of (de) activation. A review and some speculations. Journal of Psychophysiology, 3, 229–244.

[c30] Galley N. (1993). The evaluation of the electrooculogram as a psychophysiological measuring instrument in the driver study of driver behaviour. Ergonomics, 36, 1063–1070. 10.1080/00140139308967978 8404835

[c31] Galley N., & Andres G. (1996). Saccadic eye movements and blinks during long-term driving on the autobahn with minimal alcohol ingestion. Vision in Vehicles, 5, 381–388.

[c32] Harbluk J. L., Noy Y. I., Trbovich P. L., & Eizenman M. (2007). An on-road assessment of cognitive distraction: Impacts on drivers’ visual behavior and braking performance. Accident Analysis and Prevention, 39, 372–379. 10.1016/j.aap.2006.08.013 17054894

[c33] Hayhoe M., & Ballard D. (2005). Eye movements in natural behavior. Trends in Cognitive Sciences, 9, 188–194. 10.1016/j.tics.2005.02.009 15808501

[c34] Hommel B. (2010). Grounding attention in action control: The intentional control of selection In Bruya B. J. (Ed.), Effortless attention: A new perspective in the cognitive science of attention and action (pp. 121–140). Cambridge, MA: MIT Press.

[c35] Humphreys G. W., Yoon E. Y., Kumar S., Lestou V., Kitadono K., Roberts K. L., & Riddoch M. J. (2010). The interaction of attention and action: From seeing action to acting on perception. British Journal of Psychology, 101, 185–206. 10.1348/000712609X458927 19619392

[c36] Keay L., Munoz B., Turano K. A., Hassan S. E., Munro C. A., Duncan D. D., . . . West S. K. (2009). Visual and cognitive deficits predict stopping or restricting driving: The Salisbury Eye Evaluation Driving Study (SEEDS). Investigative Ophthalmology & Visual Science, 50, 107–113. 10.1167/iovs.08-2367 18719088 PMC2633220

[c37] Kingstone A., Smilek D., & Eastwood J. D. (2008). Cognitive ethology: A new approach for studying human cognition. British Journal of Psychology, 99, 317–340. 10.1348/000712607X251243 17977481

[c38] Klauer S. G., Dingus T. A., Neale V. L., Sudweeks J. D., & Ramsey D. J. (2006). The impact of driver inattention on near-crash/crash risk: An analysis using the 100-car naturalistic driving study data (NHTSA Report No. DOT HS 810 594). Blacksburg, VA: Virginia Tech Transportation Institute Retrieved from http://hdl.handle.net/10919/55090

[c39] Klein R., Kingstone A., & Pontefract A. (1992). Orienting of visual attention In Rayner K. (Ed.), Eye movements and visual cognition (pp. 46–65). New York, NY: Springer 10.1007/978-1-4612-2852-3_4

[c40] Konstantopoulos P., Chapman P., & Crundall D. (2010). Driver’s visual attention as a function of driving experience and visibility. Using a driving simulator to explore drivers’ eye movements in day, night and rain driving. Accident Analysis and Prevention, 42, 827–834. 10.1016/j.aap.2009.09.022 20380909

[c41] Kountouriotis G. K., Floyd R. C., Gardner P. H., Merat N., & Wilkie R. M. (2012). The role of gaze and road edge information during high-speed locomotion. Journal of Experimental Psychology: Human Perception and Performance, 38, 687–702. 10.1037/a0026123 22060146

[c42] Land M. F. (2006). Eye movements and the control of actions in everyday life. Progress in Retinal and Eye Research, 25, 296–324. 10.1016/j.preteyeres.2006.01.002 16516530

[c43] Land M. F. (2009). Vision, eye movements, and natural behavior. Visual Neuroscience, 26, 51–62. 10.1017/S0952523808080899 19203425

[c44] Land M., Mennie N., & Rusted J. (1999). The roles of vision and eye movements in the control of activities of daily living. Perception, 28, 1311–1328. 10.1068/p2935 10755142

[c45] Land M. F., & Tatler B. W. (2009). Looking and acting: Vision and eye movements in natural behaviour. New York, NY: Oxford University Press 10.1093/acprof:oso/9780198570943.001.0001

[c46] Lappi O. (2014). Future path and tangent point models in the visual control of locomotion in curve driving. Journal of Vision, 14, 21 10.1167/14.12.21 25761280

[c47] Lee J. D. (2008). Fifty years of driving safety research. Human Factors: The Journal of the Human Factors and Ergonomics Society, 50, 521–528. 10.1518/001872008X288376 18689062

[c48] Lestina D. C., & Miller T. R. (1994). Characteristics of crash-involved younger drivers In 38th Annual proceedings of the association for the advancement of automotive medicine (pp. 425–437). Des Plaines, IL: Association for the Advancement of Automotive Medicine.

[c49] Mackenzie A. K., & Harris J. M. (2015). Eye movements and hazard perception in active and passive driving. Visual Cognition, 23, 736–757. 10.1080/13506285.2015.1079583 26681913 PMC4673545

[c50] Mackie M.-A., Van Dam N. T., & Fan J. (2013). Cognitive control and attentional functions. Brain and Cognition, 82, 301–312. 10.1016/j.bandc.2013.05.004 23792472 PMC3722267

[c51] Mesken J., Lajunen T., & Summala H. (2002). Interpersonal violations, speeding violations and their relation to accident involvement in Finland. Ergonomics, 45, 469–483. 10.1080/00140130210129682 12167202

[c52] Moors A., & De Houwer J. (2006). Automaticity: A theoretical and conceptual analysis. Psychological Bulletin, 132, 297–326. 10.1037/0033-2909.132.2.297 16536645

[c53] Mourant R. R., & Rockwell T. H. (1972). Strategies of visual search by novice and experimental drivers. Human Factors: The Journal of the Human Factors and Ergonomics Society, 14, 325–335. 10.1177/001872087201400405 5054829

[c54] Oksama L., & Hyönä J. (2016). Position tracking and identity tracking are separate systems: Evidence from eye movements. Cognition, 146, 393–409. 10.1016/j.cognition.2015.10.016 26529194

[c55] Posner M. I., & Fan J. (2008). Attention as an organ system In Pomerantz J. R. (Ed.), Topics in integrative neuroscience (pp. 31–61). New York, NY: Cambridge University Press.

[c56] Recarte M. A., & Nunes L. M. (2003). Mental workload while driving: Effects on visual search, discrimination, and decision making. Journal of Experimental Psychology: Applied, 9, 119–137. 10.1037/1076-898X.9.2.119 12877271

[c57] Roca J., Crundall D., Moreno-Ríos S., Castro C., & Lupiáñez J. (2013). The influence of differences in the functioning of the neurocognitive attentional networks on drivers’ performance. Accident Analysis and Prevention, 50, 1193–1206. 10.1016/j.aap.2012.09.032 23084094

[c58] Savage S. W., Potter D. D., & Tatler B. W. (2013). Does preoccupation impair hazard perception? A simultaneous EEG and Eye Tracking study. Transportation Research Part F: Traffic Psychology and Behaviour, 17, 52–62. 10.1016/j.trf.2012.10.002

[c59] Schmidt D., Abel L. A., Dell’Osso L. F., & Daroff R. B. (1979). Saccadic velocity characteristics: Intrinsic variability and fatigue. Aviation, Space, and Environmental Medicine, 50, 393–395. 464963

[c60] Schneider W. X., & Deubel H. (2002). Selection-for-perception and selection-for-spatial-motor-action are coupled by visual attention: A review of recent findings and new evidence from stimulus-driven saccade control. Attention and Performance XIX: Common Mechanisms in Perception and Action, 19, 609–627.

[c61] Steinman R. (2003). Gaze control under natural conditions. Visual Neuroscience, 2, 1339–1356.

[c62] Tatler B. W., Hayhoe M. M., Land M. F., & Ballard D. H. (2011). Eye guidance in natural vision: Reinterpreting salience. Journal of Vision, 11, 5 10.1167/11.5.5 PMC3134223 21622729

[c63] Thornton I. M., Bülthoff H. H., Horowitz T. S., Rynning A., & Lee S.-W. (2014). Interactive multiple object tracking (iMOT). PLoS ONE, 9, e86974 10.1371/journal.pone.0086974 24498288 PMC3911935

[c64] Thornton I. M., & Horowitz T. S. (2015). Does action disrupt multiple object tracking (MOT)? Psihologija, 48, 289–301. 10.2298/PSI1503289T

[c65] Tomasi D., Chang L., Caparelli E. C., & Ernst T. (2007). Different activation patterns for working memory load and visual attention load. Brain Research, 1132, 158–165. 10.1016/j.brainres.2006.11.030 17169343 PMC1831676

[c66] Underwood G. (2007). Visual attention and the transition from novice to advanced driver. Ergonomics, 50, 1235–1249. 10.1080/00140130701318707 17558667

[c67] Underwood G., Chapman P., Bowden K., & Crundall D. (2002). Visual search while driving: Skill and awareness during inspection of the scene. Transportation Research Part F: Traffic Psychology and Behaviour, 5, 87–97. 10.1016/S1369-8478(02)00008-6

[c68] Underwood G., Chapman P., Brocklehurst N., Underwood J., & Crundall D. (2003). Visual attention while driving: Sequences of eye fixations made by experienced and novice drivers. Ergonomics, 46, 629–646. 10.1080/0014013031000090116 12745692

[c69] Underwood G., Crundall D., & Chapman P. (2002). Selective searching while driving: The role of experience in hazard detection and general surveillance. Ergonomics, 45, 1–12. 10.1080/00140130110110610 11964191

[c70] Underwood G., Crundall D., & Chapman P. (2011). Driving simulator validation with hazard perception. Transportation Research Part F: Traffic Psychology and Behaviour, 14, 435–446. 10.1016/j.trf.2011.04.008

[c71] Weaver B., Bédard M., McAuliffe J., & Parkkari M. (2009). Using the Attention Network Test to predict driving test scores. Accident Analysis and Prevention, 41, 76–83. 10.1016/j.aap.2008.09.006 19114140

[c72] Wickens C. D. (2002). Multiple resources and performance prediction. Theoretical Issues in Ergonomics Science, 3, 159–177. 10.1080/14639220210123806

[c73] Wickens C. D. (2008a). Multiple resources and mental workload. Human Factors: The Journal of the Human Factors and Ergonomics Society, 50, 449–455. 10.1518/001872008X288394 18689052

[c74] Wickens C. D. (2008b). Situation awareness: Review of Mica Endsley’s 1995 articles on situation awareness theory and measurement. Human Factors: The Journal of the Human Factors and Ergonomics Society, 50, 397–403. 10.1518/001872008X288420 18689045

[c75] Wickens C. D., & Hollands J. G. (2000). Engineering psychology and human performance (3rd ed.). Upper Saddle River, NJ: Prentice Hall.

[c76] Zelinsky G. J., & Neider M. B. (2008). An eye movement analysis of multiple object tracking in a realistic environment. Visual Cognition, 16, 553–566. 10.1080/13506280802000752

